# Coumarin: a novel player in microbial quorum sensing and biofilm formation inhibition

**DOI:** 10.1007/s00253-018-8787-x

**Published:** 2018-02-01

**Authors:** F. Jerry Reen, José A. Gutiérrez-Barranquero, María L. Parages, Fergal O´Gara

**Affiliations:** 1 School of Microbiology, University College Cork, Cork, Ireland; 2 BIOMERIT Research Centre, School of Microbiology, University College Cork, Cork, Ireland; 3 Instituto de Hortofruticultura Subtropical y Mediterránea La Mayora (IHSM-UMA-CSIC), Departamento de Microbiología, Facultad de Ciencias, Universidad de Málaga, Málaga, Spain; 4 Departamento de Ecología, Facultad de Ciencias, Universidad de Málaga, 29071 Málaga, Spain; 5 Human Microbiome Programme, School of Biomedical Sciences, Curtin Health Innovation Research Institute (CHIRI), Curtin University, Perth, WA Australia

**Keywords:** Coumarin, Quorum sensing inhibition, Antibiofilm, Anti-infectives, Natural products

## Abstract

Antibiotic resistance is a growing threat worldwide, causing serious problems in the treatment of microbial infections. The discovery and development of new drugs is urgently needed to overcome this problem which has greatly undermined the clinical effectiveness of conventional antibiotics. An intricate cell-cell communication system termed quorum sensing (QS) and the coordinated multicellular behaviour of biofilm formation have both been identified as promising targets for the treatment and clinical management of microbial infections. QS systems allow bacteria to adapt rapidly to harsh conditions, and are known to promote the formation of antibiotic tolerant biofilm communities. It is well known that biofilm is a recalcitrant mode of growth and it also increases bacterial resistance to conventional antibiotics. The pharmacological properties of coumarins have been well described, and these have included several that possess antimicrobial properties. More recently, reports have highlighted the potential role of coumarins as alternative therapeutic strategies based on their ability to block the QS signalling systems and to inhibit the formation of biofilms in clinically relevant pathogens. In addition to human infections, coumarins have also been found to be effective in controlling plant pathogens, infections in aquaculture, food spoilage and in reducing biofouling caused by eukaryotic organisms. Thus, the coumarin class of small molecule natural product are emerging as a promising strategy to combat bacterial infections in the new era of antimicrobial resistance.

## Introduction

The indiscriminate use of antibiotics has brought us to the brink of a new era where the emergence of multidrug-resistant bacterial and fungal pathogens poses one of the major health issues of global concern (Fernandez and Hancock 2012; Tanwar et al. 2014). The rapid decline in the discovery of novel antibiotics in the last two decades has coincided with the emergence of resistance to all known classes of clinically used antibiotics. While the urgent need to discover and develop novel strategies to combat microbial infections is recognised, how this is best achieved is less clear. The case for growth-limiting antibiotics remains strong, and decorated derivatives of existing antibiotics continue to make it to the clinic. However, with the possible exception of the newly discovered teixobactin (Ling et al. 2015), resistance to newly introduced antibiotics is likely to emerge in the short rather than the long term. A particularly notable case is the recent reports of resistance to the polymyxin class of antibiotic, long regarded a salvage therapy for which transmissible resistance was unlikely to emerge. This was largely due to the fact that polymyxins target the cell membrane, and therefore plasmid-encoded resistance would be more difficult to achieve. However, reports from China and the USA have confirmed the transmission of plasmid-encoded resistance among *Escherichia coli* isolates (Liu et al. 2016).

Owing largely to the challenges associated with antibiotic resistance and the dearth of new antibiotics in the development pipeline, researchers have pursued alternative strategies to manage microbial infections. One of the best developed of these are strategies that target a microbial signalling system termed quorum sensing (QS) (Cegelski et al. 2008; Cooper and Shlaes 2011; LaSarre and Federle 2013). QS is a cell to cell communication signalling system that controls the expression of, in some cases, hundreds of genes related to virulence phenotypes in clinical human pathogens (Papenfort and Bassler 2016). QS regulons are known to vary between species, as indeed does the spectrum of virulence-related phenotypes that falls under QS control (Whiteley et al. 2017). Biofilm formation is one of the most important virulence phenotypes of opportunistic human pathogens, and it is under the control of the QS system in several important pathogenic organisms. A structured community of bacteria that requires intact cell-cell communication for its initiation and maturation, the biofilm lifestyle poses a significant challenge to the effectiveness of conventional antibiotics and is considered a breeding ground for antibiotic resistance (Hoiby et al. 2011). In recent years, increasing attention has been paid to finding novel therapeutic strategies specifically targeting QS signalling systems and the biofilm mode of growth, providing the pillar upon which the future of next-generation antivirulence therapies would be forged (de la Fuente-Nunez et al. 2014; Kalia 2013; Njoroge and Sperandio 2009). Sourcing these compounds has followed quite diverse paths with bioprospecting of natural ecosystems and synthetic remodelling occurring separately and as integrated endeavours. Plant phenolics have delivered a broad range of bioactive compounds, many of which are now being considered for their anti-infective potential.

The bioactive potential of different environments and their associated organisms have been established over the last number of decades, with soil and more recently marine life proving to be a rich source of novel compounds that may be exploited as QS inhibitors and antibiofilm agents (Gutierrez-Barranquero et al. 2017; Kalia 2013; Manefield et al. 2002; Miquel et al. 2016; Rabin et al. 2015; Rasmussen et al. 2005; Sayem et al. 2011). Plant secondary metabolites, and specifically plant phenolic compounds (Slobodnikova et al. 2016), have been widely used for decades because of their great pharmacological properties (Venugopala et al. 2013). Coumarins are a large family of naturally derived fused benzene and a-pyrone rings found primarily in a wide range of plant sources. Some coumarins are regarded as phytoalexins which are plant resistance compounds that are biosynthesised by plant tissues in response to pathogenic infection (Yang et al. 2017). Members of the coumarin class of compound have also been identified in bacteria and fungi, such as novobiocin and coumermycin isolated from *Streptomyces* (Eustaquio et al. 2003), and aflatoxins isolated from different *Aspergillus* species (Kumar et al. 2016).

**Fig. 1:**
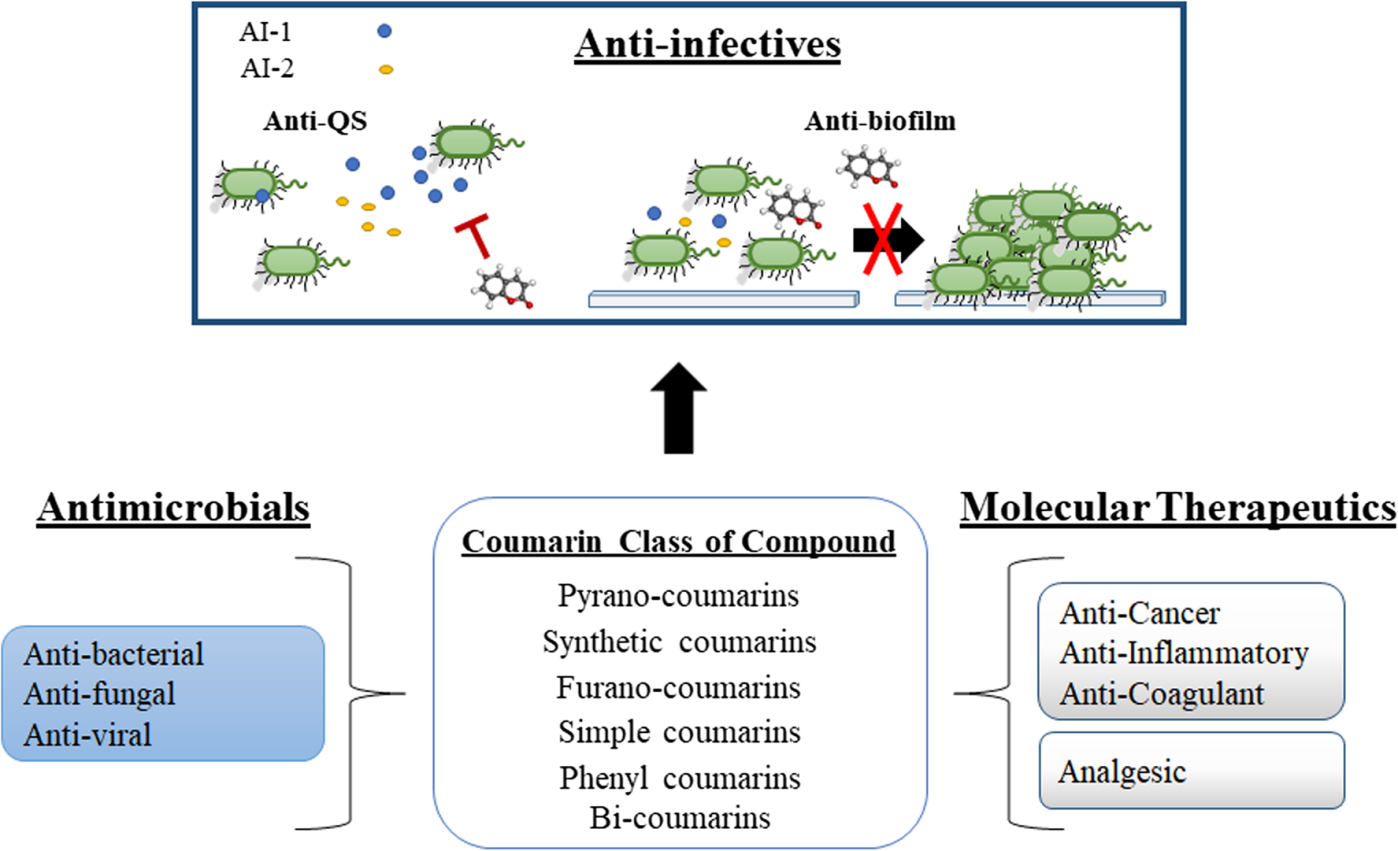
The coumarin class of plant phenolic compound have been shown to possess several important pharmacological properties. More recently, a role in the modulation of microbial behaviour has emerged, with several reports describing interference with cell-cell communication (quorum sensing) and the formation of multicellular microbial structures (biofilms). Particular emphasis has been placed on the ability of coumarins to disrupt AI-1 and AI-2 signalling in a range of important microbial pathogens

The role of coumarins as antimicrobial molecules has been extensively studied to date (Al-Majedy et al. 2016) with compounds having long chain hydrocarbon substitutions being particularly active (Venugopala et al. 2013). Among many other relevant therapeutic activities, coumarins are notable for their role as anticancer, anti-inflammatory, antimicrobial, anti-oxidant and anticoagulant bioactive compounds (Joubert et al. 2017; Kapp et al. 2017; Mandlik et al. 2016; Venugopala et al. 2013). Structurally, these naturally produced coumarins comprise a diverse spectrum of modifications from the parent molecule (Murray 2002), with pyrano- and furano-coumarins being particularly pharmacologically active. However, somewhat surprisingly, the parent molecule coumarin possesses very low antibacterial activity, in contrast to its decorated counterparts. Rather than being biologically inactive, it would appear that coumarin plays a more subtle role in the natural ecosystem, one where governance of microbial behaviour is controlled through suppression of QS signalling and the formation of biofilms (Fig. 1). Recent reports on the role of coumarins as QS inhibitors and antibiofilm agents (Gutierrez-Barranquero et al. 2015; Lee et al. 2014) have drawn the attention of many research groups to the potential of coumarin as a natural non-toxic anti-infective. This review provides an insight into the bioactivity of the coumarin class of plant phenolics with particular emphasis on the emerging role of coumarin as a novel QS inhibitor and antibiofilm agent across a broad spectrum of microbial pathogens.

### Coumarins from diverse ecological sources possess potent antimicrobial activity

Plant extracts have long been known to possess beneficial activities for human health, and have been used since ancient times as medicines, particularly in Oriental cultures. Although the basis of these health-promoting properties is still only partly understood, the effectiveness of plant extracts as sources of lead compounds for modern drug development underpins the importance of these natural sources of bioactive compounds. Apart from the development of potent therapeutics for the treatment of cancer, inflammation and other clinical conditions, plant extracts have also provided a range of compounds that target microbial pathogens. The coumarin class of phenolic compound derived from plant extract have received considerable attention based on their antimicrobial properties, and are emerging as a promising candidate for next generation antimicrobial development.

The antibacterial and anti-oxidant activities of several coumarins and pyranocoumarins isolated from the roots of *Ferulago campestris* were reported against gram-negative and gram-positive pathogens. These included *Staphylococcus aureus*, *Salmonella enterica* serovar Typhi, *Enterobacter cloacae* and *Enterobacter aerogenes* (MIC 16–32 μg/mL) as well as the digestive tract pathogen *Helicobacter pylori* (MIC 5–25 μg/mL) (Basile et al. 2009). Similarly, *Prangos hulusii* root extracts yielded a new prenylated coumarin in addition to nine that were previously characterised. The root extract and its prenylated coumarins were found to exhibit antimicrobial activity against clinical pathogens including *Bacillus subtilis* and *Klebsiella pneumoniae* (MIC 5–125 μg/mL) (Tan et al. 2017). Methanol extracts prepared from seven plants grown in Finland were found to produce antimicrobial activity against bacterial and fungal pathogens, although the activities of six natural coumarin compounds were reportedly weak, with the exception of the inhibitory effect against the fungal pathogen *Fusarium culmorum* (Ojala et al. 2000). Three new coumarin derivatives and three new furanocoumarins were isolated from the fern *Cyclosorus interruptus* (Willd.) H. Itô (Quadri-Spinelli et al. 2000). One of the former and two of the latter were found to possess antibacterial activity. An aryl coumarin glucoside, asphodelin A 4’-*O*-β-D-glucoside, isolated from *Asphodelus microcarpus* exhibited antibacterial activity against *S. aureus*, *E. coli* and *Pseudomonas aeruginosa* as well as modest antifungal activity against the fungal pathogens *Candida albicans* and *Botrytis cinerea* (El-Seedi 2007)*.* Interestingly, the bulbs and roots of *A. microcarpus* have long been used by Bedouin tribes as an antimicrobial agent. Kayser and Kolodziej working with simple coumarins described broad diversity regarding growth inhibitory activity with minimum inhibitory concentrations ranging from 0.9 to > 12.4 μM (Kayser and Kolodziej 1999). Pathogens tested included *S. aureus,* beta-hemolytic *Streptococcus, Streptococcus pneumoniae, E. coli, K. pneumoniae, P. aeruginosa, Proteus mirabilis* and *Haemophilus influenzae*. While coumarins with a methoxy function at C-7 and, if present, an OH group at either the C-6 or the C-8 position were invariably effective, the presence of an aromatic dimethoxy arrangement was found to be favourable against those microorganisms which require special growth factors (beta-haemolytic *Streptococcus*, *S. pneumoniae* and *H. influenzae*). However, in spite of the broad range of activity of coumarin compounds, the simple coumarin structure itself has very low antibacterial activity. The reason for this loss of activity in the absence of long hydrocarbon chains or other decorations is not yet understood. However, recent reports have suggested that, rather than limiting the growth of microbial pathogens, coumarin itself can target a key microbial cell-cell communication system and with it, the ability to inhibit antibiotic tolerant colonisation structures called biofilms.

## Coumarins as anti-QS and antibiofilm agents against clinically relevant pathogens

Coumarins were first considered as potential anti-QS and antibiofilm compounds following a virtual QS inhibitor screen of a Traditional Chinese Medicine library by docking analysis against the *Agrobacterium tumefaciens* QS transcriptional activator protein TraR (Zeng et al. 2008). This study revealed that esculetin (6,7-dihydroxycoumarin) and esculin were structurally compatible with the TraR signal binding site. Although this study did not explore the QS inhibitory function experimentally, the authors did demonstrate that both coumarin derivatives were able to inhibit the *P. aeruginosa* biofilms at a concentration of 200 μM. In a separate study, Brackman and colleagues showed that both of these molecules have a moderate ability to suppress the biofilm formation of two species of the *Burkholderia cepacia* complex (Brackman et al. 2009). Moreover, in this case, the authors were able to demonstrate inhibition of QS signalling using biosensor reporter strains (Brackman et al. 2009). Two furocoumarins isolated from grapefruit juice, bergamottin and dihydroxybergamottin, were able to suppress QS biofilm formation in *E. coli* O157:H7 up to 72 and 58.3%, respectively (Girennavar et al. 2008). Antibiofilm activity of these compounds against *S. enterica* serovar Typhimurium (15.5 and 46.5%, respectively) and *P. aeruginosa* (18.1 and 27.3%, respectively) was more modest (Girennavar et al. 2008). In this latter study, the authors demonstrated how both furocoumarins were able to inhibit AI-1 (*N*-acyl homoserine lactones, AHLs) and AI-2 (furanosyl borate diester) signalling using Tn5 mutants of *Vibrio harveyi* as reporter strains. Following this, an interesting study conducted by Durig et al. (2010) using a chemo-informatic approach targeting the Chinese natural product database (CNPD) developed a series of 2nd and 3rd generation compounds from ellagic acid using a coumarin scaffold. Esculetin (2nd generation) was found to be a potent antibiofilm agent towards *S. aureus* strain 8324, but did not affect biofilm formation of *S. aureus* NCTC4671 or ATCC 27957. The 3rd generation compound fisetin was shown to be active against all three strains (Durig et al. 2010). A virtual docking approach also identified nodakenetin and fraxin (two coumarin compounds extracted from *Peucedanum decursivum* (Miq). Maxim and *Fraxinus chinensis* Roxb., respectively) as putative QS inhibitors, with the antibiofilm ability of both subsequently demonstrated in *Pseudomonas aeruginosa* and to a lesser extent *Stenotrophomonas maltophilia* (Ding et al. 2011).

More recently, attention has switched to the simple coumarin compound, with several studies revealing a broad spectrum of antivirulence activity. Lee et al. (2014) carried out a screen of 560 phytochemicals to identify new antibiofilm compounds against *E. coli* O157:H7. Restricting the second round of screening only to hits where the biofilm reduction was above 70%, only four compounds met the requirement, one of them being a sesquiterpene coumarin (Lee et al. 2014). Following this, the authors subsequently investigated the role of eight different coumarins [coumarin, coumarin-3-carboxylic acid, dephnetin, ellagic acid, esculetin, 4-hydroxycoumarin, scopoletin and umbelliferone (7-hydroxycoumarin)] for antibiofilm activity against the *E. coli* O157:H7 strain. Similar to coladonin, coumarin and umbelliferone showed the highest biofilm inhibition, reaching values of 80 and 90%, respectively. qRT-PCR analysis revealed that neither compound impacted on AI-2 signalling, with expression of the *luxS* gene encoding the synthase responsible for AI-2 production being unaffected in the presence of either coumarin or umbelliferone. However, expression of the QS-controlled *lsrA* gene was decreased in response to either compound. As such, the anti-QS activity of these coumarin compounds remains to be determined, as does its role if any in the biofilm suppression activity of coumarins against this important human pathogen.

**Fig. 2:**
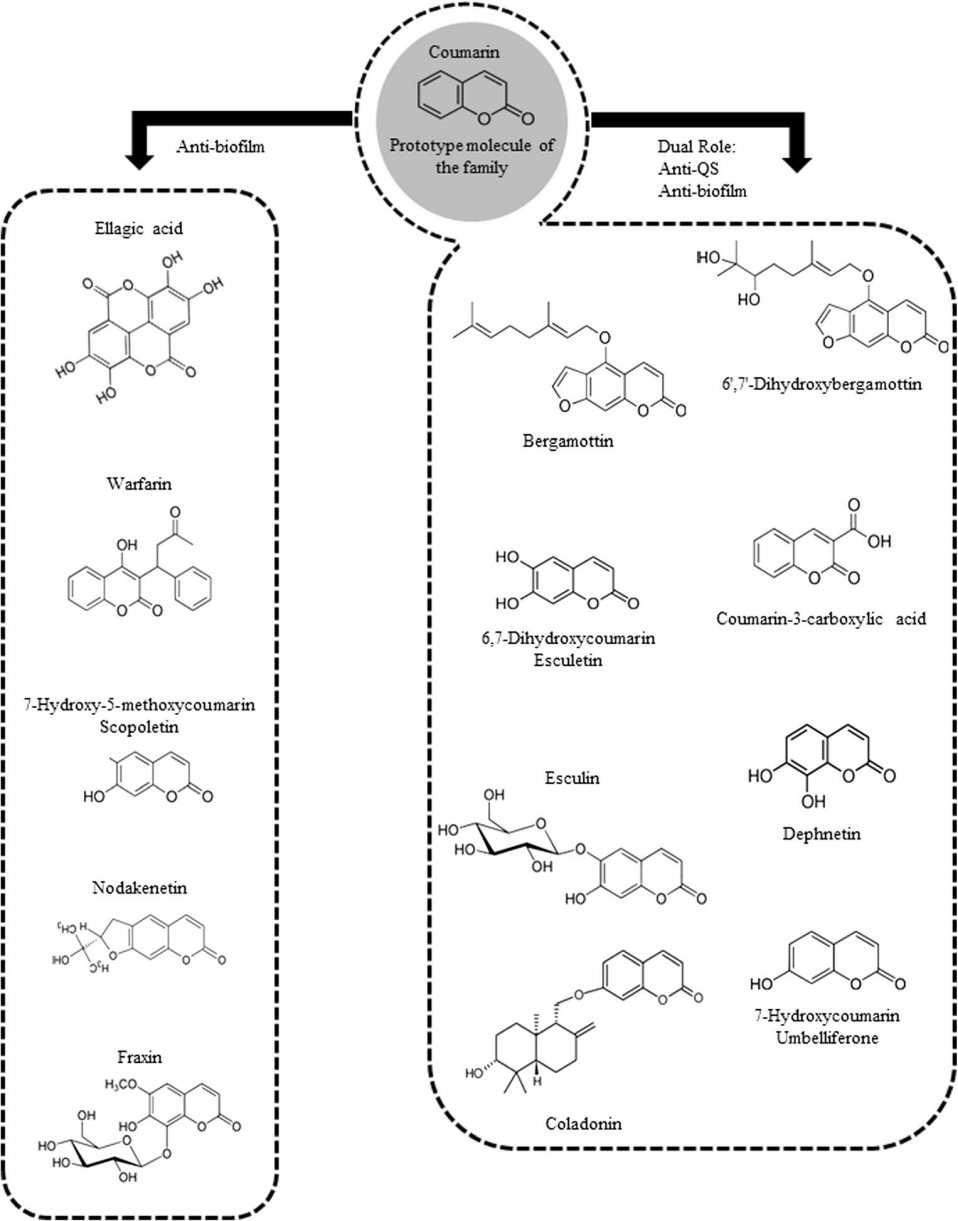
A schematic overview of coumarin structures and their anti-infective properties. Compounds for which anti-biofilm properties have been established are presented on the left, while those with dual activity are presented on the right

Confirmation of the QSI activity of coumarin came from a study by our own group in 2015 where we analysed the QS mode of action of coumarin with a combination of direct and signal competition biosensor assays (Gutierrez-Barranquero et al. 2015). The QS inhibition biosensor reporter strains *Serratia marcescens* SP15, *Chromobacterium violaceum* DSM 30191 *A. tumefaciens* NTL4 all showed pigment inhibition in the presence of increasing concentrations of coumarin. These findings were independent of any growth-related effects. In addition, signal competition assays using the QS biosensor reporter strains *S. marcescens* SP19, *C. violaceum* CV026 and *A. tumefaciens* NTL4 (adding specific AHLs) suggested an AHL-specific interference. In order to further establish the QSI activity of coumarin, and to determine whether or not it extended to a clinically relevant pathogen, *P. aeruginosa* PA14 was selected for analysis. Coumarin decreased the expression of the QS-related genes *pqsA* and *rhlI* in *Pseudomonas aeruginosa* PA14. Coumarin also inhibited biofilm formation in this pathogen, along with several other virulence phenotypes including swarming motility and phenazine production. The broad-spectrum activity of coumarin was revealed by demonstrating inhibition of biofilm formation of other gram-negative and gram-positive bacteria (*E. coli*, *Edwardsiella tarda*, *Vibrio anguillarum* and *S. aureus*), protease activity (*S. maltophilia* and *B. cepacia*) and bioluminescence (*Allivibrio fischeri*). Taken together, these data support a role for coumarin in the suppression of QS and biofilm formation in clinically relevant pathogens (Fig. 2). However, further mechanistic studies will be required before coumarin can be considered a universal QS and biofilm inhibitor. This is particularly true in light of the multitude of QS systems in different bacterial species affected and the current lack of molecular mechanism to explain the possible anti-QS and antibiofilm effects of coumarin.

Warfarin an important coumarin derivative that is widely used as an oral anticoagulant agent worldwide (Pirmohamed 2006) was tested for antibiofilm activity against *E. coli* and found to reduce biofilm formation by 40% when tested at 7.5 mM (Ojima et al. 2016). A lower concentration of 5 mM warfarin did not significantly impact biofilm formation (Ojima et al. 2016). In the same study, lactoferrin (a milk protein that shows a wide range of biological properties, including antimicrobial function) and ampicillin (a β-lactam antibiotic) were shown to promote biofilm formation in *E. coli* when administered at subinhibitory concentrations. Interestingly, the authors demonstrated that in the presence of either compound, warfarin significantly decreased biofilm formation of *E. coli* by 50% at a concentration of 5 mM. This study adds another layer of complexity to the mode of action of coumarins, supporting the idea that some could impact biofilm formation promoted by subinhibitory concentrations of other molecules.

### Non-clinical biotechnological applications of coumarin QSI and antibiofilm activity

As might be expected, the role of coumarins in QSI and biofilm formation inhibition has been typically tested in human bacterial pathogens that cause serious health problems in hospitals. However, recent studies have also exploited the anti-QS and antibiofilm properties of coumarins in other systems.

Bacterial wilt a disease elicited by *R. solanacearum* produces severe economic losses to important crops such as tomato, potato, tobacco and eggplant in tropical and subtropical regions worldwide. As coumarins have been reported previously for their antibacterial activities (Barot et al. 2015), 18 different coumarin derivatives including some hydroxylated variations were tested for antibacterial and antibiofilm activity against *R. solanacearum* (Yang et al. 2016). From the 18 coumarin tested, 4 of them showed the highest biofilm formation inhibition, coumarin, and three different hydroxycoumarins (umbelliferone, esculetin and dephnetin). It is worth noting that the best antimicrobial activities were also displayed by these same coumarins, raising the possibility that the biofilm suppression may simply reflect the limitation of growth under these conditions. The authors subsequently followed up this study with analysis of umbelliferone and its antivirulence activity against *R. solanacearum* (Yang et al. 2017). The type three secretion system (T3SS) and biofilm formation were both suppressed.

Marine biofouling is the accumulation of micro- and macro-organisms on submerged structures in seawater. Currently, it is a persistence problem that causes severe economic losses and also has an impact from an ecological point of view (Callow and Callow 2011). A recent study described the role of 7-hydroxy-4-methylcoumarin as an effective agent against biofouling, inhibiting both the settlement as well as the byssogenesis of mussels (Perez et al. 2016). Although the role of coumarin as a novel QS inhibitor has been demonstrated previously (Gutierrez-Barranquero et al. 2015), there was no attempt to explore whether coumarin could be used as a protective treatment in aquaculture. In this sense, Zhang and coworkers (2017) analysed the protective role of coumarin in relation to infection by *Vibrio splendidus* in an aquaculture model of *Apostichopus japonicas* (Zhang et al. 2017). The authors observed that coumarin could interfere with the induction of QS-regulated virulence genes by cell-free supernatants and ethyl acetate extracts of *V. splendidus* shown to possess AHLs by activation of *C. violaceum* CV026. Thus, they proposed that coumarin could be used as a QS inhibitor to possibly modulate *V. splendidus* infections in future aquaculture applications. The authors also reported that coumarin did not exhibit any effect on biofilm formation, possibly due to the fact that *V. splendidus* showed poor ability to develop in vitro biofilms on polystyrene microtitre plates (Zhang et al. 2017). Finally, Hou and coworkers analysed the ability of dihydrocoumarin to inhibit the biofilm formation of *Hafnia alvei*, a bacterium that is mainly found in spoiled food (Hou et al. 2017). Biofilm formation in this organism has previously been linked to QS, although its contribution to persistence and spoilage per se remains to be fully elucidated (Viana et al. 2009). The authors demonstrated the capacity of dihydrocoumarin in the inhibition of violacein production by *C. violaceum* CV026, and also, observed a high impact on the reduction of biofilm formation at approximately 90%. These findings indicate that dihydrocoumarin could be a useful QS inhibitor or antibiofilm agent for potential development in controlling food spoilage organisms (Bai and Rai 2011).

## Structure-function analysis of the QSI and antibiofilm activity of coumarins

Previous studies had investigated structure-function relationships of coumarins linked to antibacterial activity. As coumarin has been identified as a potent antivirulence molecule, and because different substitutions in specific sites of the coumarin molecule can affect its biological activity (Barot et al. 2015), a SAR analysis was a logical undertaking in relation to understanding QSI and antibiofilm activity. D'Almeida et al. (2017) followed such an approach to test if the different substitutions on the coumarin structure could affect the QSI activity (D'Almeida et al. 2017). Using *P. aeruginosa* and *C. violaceum* as test species, D’Almeida and colleagues (D'Almeida et al. 2017) performed a comparison of seven structurally related coumarins (coumarin and different hydroxylated derivatives). Initially using a bioassay with the QS biosensor *C. violaceum* CV026, and adding exogenous hexanoyl homoserine lactone (HHL), the authors demonstrated that all the different coumarins tested, with the exception of 4-hydroxycoumarin and dihydrocoumarin, were able to inhibit the violacein production. In a follow-up test using *C. violaceum* ATCC 12472, the authors observed that both 4-hydroxycoumarin and dihydrocoumarin were indeed able to reduce the percentage of violacein production, although to a lesser extent compared to the other coumarins tested. Finally, the authors tested the capacity of the different coumarins to inhibit biofilm formation of *P. aeruginosa.* Esculetin, umbelliferone and coumarin showed the highest *P. aeruginosa* biofilm inhibition, while 4-hydroxycorumarin and dihydrocoumarin presented the lowest activity in this regard. This contrasts with the relatively low antibiofilm activity of esculetin towards *E. coli* described by Lee et al. (2014), and suggests that species or strain heterogeneity may play some role in this response. The low activity associated with hydroxylated coumarins has previously been reported in *E. coli* with respect to biofilm formation (Lee et al. 2014). Hydroxylation at position 4 or position 8 (dephnetin) of the coumarin structure was found to dramatically diminish antibiofilm activity while the same modification at position 7 led to enhanced activity. Di-hydroxylation of coumarin at positions 6 and 7 (esculetin) led to a reduction in activity compared to simple coumarin in this study. Replacing the 6-hydroxy group with a 6-methoxy group (scopoletin) did not affect antibiofilm activity, while decoration at position 3 with a carboxy group diminished activity compared with the parent compound (Lee et al. 2014). The introduction of a sesquiterpene in position 7 (coladonin) neither enhanced nor diminished activity with respect to coumarin.

Structural modifications of the coumarin framework have also been undertaken with the aim of developing more effective pharmacologically active coumarins (Saleem et al. 2010). Several synthetic coumarins with a variety of pharmacophoric groups at C-3, C-4 and C-7 positions have been intensively screened for antimicrobial, anti-HIV, anticancer, lipid-lowering, anti-oxidant and anticoagulation activities (Kulkarni et al. 2006). Hydroxylation of coumarins at the C-6, C-7 or C-8 position was shown to significantly enhance the antibacterial activity against *R. solanacearum* (Yang et al. 2016). Guan and coworkers showed that decoration of the coumarin framework with methoxyacrylate moieties derived from natural strobilurin A can generate possible lead compounds for developing novel fungicides (Guan et al. 2011). Potent antimicrobial and anti-inflammatory activities have been described for C4-substituted aryloxymethyl, arylaminomethyl and dichloroacetamidomethyl coumarins, along with the corresponding 1-azacoumarins (Kulkarni et al. 2006). Kalkhambkar and colleagues reported the synthesis of a series of new fluorinated coumarins and 1-aza coumarins (Kalkhambkar et al. 2008). Introduction of fluorine at the 4′-position in the aryloxy and arylamino moieties of both coumarin and 1-aza-coumarin was found to enhance the antimicrobial properties of the compounds. Analgesic and anti-inflammatory properties were also enhanced in these modified compounds. More recently, a series of novel coumarin–benzimidazole hybrids were designed and synthesised. Compounds showed broad-spectrum antibacterial activity against *P. aeruginosa*, *S. aureus*, *Bacillus subtilis* and *P. vulgaris* (Singh et al. 2017).

## Conclusion and future challenges

The importance of developing new anti-infective compounds with a real possibility of clinical use is underscored by recent reports of a severe lack of pipeline compounds with antimicrobial activity. Not alone is antibiotic resistance on the increase, and the discovery of new antibiotics long since passed into rapid decline, but the introduction of new chemical entities has also plateaued (Reen et al. 2015). Therefore, there is an urgent need to develop innovative new compounds that can either (i) directly address the threat of superbugs and resistant pathogens or (ii) enable conventional antibiotics to do so. Over the last decade, coumarins have received considerable attention in this regard, being isolated from natural sources, chemically decorated or synthetically derived. Apart from exhibiting antibacterial, antifungal and antiviral activity, several members of the coumarin class of compound target cell-cell signalling through QSI, and the aggregation of multicellular communities of biofilms (summarised in Table 1). Future studies will no doubt focus on improved properties from the perspective of increased bioactivity and clinical compatibility. However, understanding the role of these compounds in their natural ecosystem may also uncover new applications for this biotechnologically important class of molecule.

**Table 1:** Coumarin targets QS and biofilm formation in pathogenic bacteria

Coumarin compound	Target organism	Conc (μg/ml)	QSI	Antibiofilm	Reference
Coumarin	*E. coli* O157:H7	50	*lsrA*	**+++**	(Lee et al. 2014)
Umbelliferone	*E. coli* O157:H7	50	*lsrA*	**+++**	
Coladonin	*E. coli* O157:H7	50		**+++**	
Coumarin-3-carboxylic acid	*E. coli* O157:H7	50		**++**	
Dephnetin	*E. coli* O157:H7	50		**++**	
Ellagic acid	*E. coli* O157:H7	50		**++**	
Esculetin	*E. coli* O157:H7	50		**++**	
4-Hydroxycoumarin	*E. coli* O157:H7	50		**++**	
Scopoletin	*E. coli* O157:H7	50		**++**	
Coumarin	*P. aeruginosa*	200	*rhl, pqsA*	++	(Gutierrez-Barranquero et al. 2015)
Coumarin	*Edwardsiella tarda*	200		+	
Coumarin	*E. coli* MUH	200		++	
Coumarin	*S. aureus* NCDO949	200		++	
Coumarin	*V. anguillarum*	200		++	
Coumarin	*A. fischeri*	200	Bioluminescence		
Dihydroxybergamottin	*V. harveyii*	1	BB886 *luxP* Tn5		(Girennavar et al. 2008)
			BB170 *luxN* Tn5		
Bergamottin	*V. harveyii*	1	BB886 *luxP* Tn5		
			BB170 *luxN* Tn5		
Dihydroxybergamottin	*E. coli*	1		+++	
Bergamottin	*E. coli*	1		+++	
Dihydroxybergamottin	*S. typhimurium*	1		+	
Bergamottin	*S. typhimurium*	1		++	
Dihydroxybergamottin	*P. aeruginosa*	1		+	
Bergamottin	*P. aeruginosa*	1		++	
Esculetin	*S. aureus* 8324	128		+++	(Durig et al. 2010)
Fisetin	*S. aureus* 8324	16		+++	
Fisetin	*S. aureus* NCTC	16		+++	
Fisetin	*S. aureus* ATCC	16		+++	
Nodakenetin	*P. aeruginosa*	81		+++	(Ding et al. 2011)
Nodakenetin	*S. maltophilia*	81		++	
Fraxin	*P. aeruginosa*	74		++	
Fraxin	*S. maltophilia*	74		+	
Umbelliferone	*R. solanocearum*	50		+++	(Yang et al. 2017)
Coumarin	*V. splendidus*	985	*Vsm*, *Vsh*		(Zhang et al. 2017)
Warfarin	*E. coli*	1540		**++**	(Ojima et al. 2016)

From the perspective of understanding the ecological role of coumarins, focus and attention will naturally veer towards the rhizosphere interactome where these compounds are thought to play a considerable role. Exuded from the roots of plants, the antibacterial and antifungal activity of coumarins is thought to affect the dynamics of the resident microbiota, and its interplay with other constituents of this rich ecosystem with high species richness. However, while we often view antimicrobials as weapons, whether this is the case in a natural ecosystem, or whether the low concentrations act as signals remains to be established. In this sense, the QSI and antibiofilm activity for coumarins, and the structural specificity that underpins this activity, could reflect a more subtle role for these compounds in situ. The aforementioned activities would enable the plant to moderate localised microbiota with a lesser threat of resistant pathogens adapting to the growth-limiting activity of its antimicrobial counterparts. It is also possible that the presence of QSI and antibiofilm compounds in the rhizosphere may increase the effectiveness of antimicrobials, restricting microbes to planktonic antibiotic susceptible growth.

The molecular mechanism by which coumarins effect their QSI and antibiofilm activity remains to be determined. While suppression of QS could explain some of the antibiofilm activity, the interactions that govern this impact remain uncharacterised. It is also worth noting that coumarins can disrupt both AI-1 and AI-2 signalling in some cases, both structurally distinct systems for which a unifying principle remains to be elucidated. From the perspective of biofilm inhibition, the ability of coumarin to interfere with biofilms in both gram-negative and gram-positive pathogens suggests that QS signalling alone is unlikely to explain its mechanism of action. In some cases, growth-limiting effects of coumarins cannot be ruled out. The coumarin concentrations used in these studies ranged from 1 μg/ml (Girennavar et al. 2008) to 1.5 mg/ml (Ojima et al. 2016), with limited investigation of the impact of these active compounds on the growth of target pathogens. Both biofilm and QS are cell density-dependent multicellular phenotypes, and as such can be significantly altered by reductions in growth rate or biomass. Therefore, growth kinetic assays and dose dependency curves will be needed to establish the growth independence and true QS and biofilm inhibitory activity of these compounds. Furthermore, while some of the studies cited used analytical technologies such as liquid chromatography mass spectrometry and thin layer chromatography for QS detection, several studies relied solely on activation or suppression of pigment production in classical biosensor strains. Although widely used for the identification of QS producing/inhibiting isolates, off-target effects have been reported, highlighting the need for confirmatory analysis (Defoirdt et al. 2013). Therefore, it is clear that further studies on the mechanism are needed to support the ongoing development of derivative compounds towards clinical use.

From a pharmacological perspective, the applicability of coumarin in clinical applications would appear to be favourable. Although liver toxicity in rodents has been reported (Felter et al. 2006), and cancerogenic activity was reported in early studies on the simple coumarin structure (Lake 1999), coumarin is not genotoxic. Therefore, exposure to coumarin in food or cosmetics products is not thought to pose any risk to humans (Lee et al. 2014). A therapeutic dose of 0.64 mg/kg of body weight is considered safe and will guide the clinical application of these compounds going forward (Felter et al. 2006). Hence, structure-function studies and chemical modifications linked to improved bioactivity will be crucial in ensuring that these compounds can be brought through clinical testing and find application in the management of infections. This is by no means a foregone conclusion and it should be noted that despite almost 20 years of research into QSI and antibiofilm compounds, the commercial route to the clinic for these promising molecules has yet to be reached. The natural product status of coumarins and their relatively benign toxicity may help to bridge this gap.
